# Denial-of-Service Attack on IEC 61850-Based Substation Automation System: A Crucial Cyber Threat towards Smart Substation Pathways

**DOI:** 10.3390/s21196415

**Published:** 2021-09-26

**Authors:** Suleman Ashraf, Mohammad H. Shawon, Haris M. Khalid, S. M. Muyeen

**Affiliations:** 1 School of Electrical Engineering Computing and Mathematical Sciences, Curtin University, Perth, WA 1987, Australia; suleman.ashraf@student.curtin.edu.au (S.A.); mhshawon@postgrad.curtin.edu.au (M.H.S.); 2 Department of Electrical and Electronics Engineering, Higher Colleges of Technology, Sharjah 27272, United Arab Emirates; harism.khalid@ieee.org; 3 Department of Electrical Engineering, Qatar University, Doha 2713, Qatar

**Keywords:** anomaly detection, cyber-attacks, cyber-attack detection, cyber grid elements, cyber threat, denial-of-service attack, intrusion detection, power systems, power system dynamics, smart grid, substation automation system, substation pathways

## Abstract

The generation of the mix-based expansion of modern power grids has urged the utilization of digital infrastructures. The introduction of Substation Automation Systems (SAS), advanced networks and communication technologies have drastically increased the complexity of the power system, which could prone the entire power network to hackers. The exploitation of the cyber security vulnerabilities by an attacker may result in devastating consequences and can leave millions of people in severe power outage. To resolve this issue, this paper presents a network model developed in OPNET that has been subjected to various Denial of Service (DoS) attacks to demonstrate cyber security aspect of an international electrotechnical commission (IEC) 61850 based digital substations. The attack scenarios have exhibited significant increases in the system delay and the prevention of messages, i.e., Generic Object-Oriented Substation Events (GOOSE) and Sampled Measured Values (SMV), from being transmitted within an acceptable time frame. In addition to that, it may cause malfunction of the devices such as unresponsiveness of Intelligent Electronic Devices (IEDs), which could eventually lead to catastrophic scenarios, especially under different fault conditions. The simulation results of this work focus on the DoS attack made on SAS. A detailed set of rigorous case studies have been conducted to demonstrate the effects of these attacks.

## 1. Introduction

**Figure 1:**
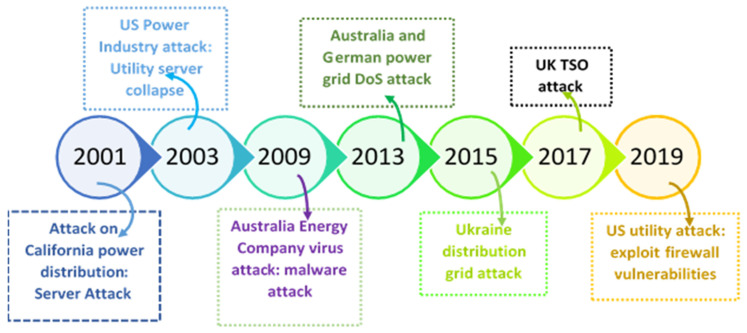
Major cyber-attacks on the electric power grid.

In this era of rapid development of modern civilization, the electrical power grid is considered as one of the most important and critical infrastructures for any country. The evolution of smart grid and the introduction of Information and Communication Technologies (ICT) have made the power grid vulnerable to cyber-attacks and other security threats. The National Institute of Standards and Technology (NIST) reports that there have been significant increases in the number of cyber-attacks on electrical power grids over the last decade [1]. One of the most notable cyber-attacks on power grid is the strike on the Ukrainian power grid in December 2015, where the hacker accessed the Supervisory Control and Data Acquisition (SCADA) information, disrupted the normal operation, and caused the disconnection of 30 substations in total, affecting 225,000 customers for approximately 3 h [2]. The major attacks on the electric power grid [3] in this millennium has been depicted in Figure 1. In this context, NIST has outlined and set the three primary cyber security requirements for the smart grid. These three requirements are: (1) availability, (2) integrity, and (3) confidentiality, respectively [4]. They are further termed as the “essential criteria for cyber security”.

In power grids, a grid substation is an integral component of the power system. The substation serves a multitude of purposes such as: (1) stepping the voltage up or down to allow transmission and distribution, (2) managing fluctuations in voltage, (3) controlling the network for maintenance, (4) providing protection from faults, and (5) allowing the power network to be managed by utilizing circuit breakers, switches, and other devices. These all purposes are well taken by the ICT-enabled SCADA systems in the present era of smart grids, which can monitor and control the grid more efficiently and autonomously. In addition to the enhanced communication technologies, the development of IEC 61850-based smart substations has allowed for better monitoring and control of the power system by utilizing Ethernet communications and IEDs [5]. However, with the process of digitalization, these substations have become increasingly more complex and have been exposed to potential cyber security threats which could lead to a shattering effect on the power system. These threats are posed from numerous parties such as hackers, ex-employees, competitors, and even maintenance personnel. Being the core part of the power grid, the security and the reliability of the power grid needs to be ensured first at the substation level [6]. Therefore, it is important to develop appropriate solutions to ensure the safety of the grid and protect it from threats from all the possible sources, which is the focus and motivation of this work.

Recently, the cyber security for substations has received much attention among the researchers [7,8]. There have been several additional standards proposed and developed by IEC such as: (1) IEC 62351 standards for the power system information infrastructure [9] and (2) IEC 62443 standards for SCADA systems and industrial control systems security [10,11]. In addition, several work reports on the techniques and methodologies to tackle cyber security issue on smart grid. For example, the intrusion detection system for IEC 61850-based smart substation has been highlighted in [12,13]. Cyber security-based behavior analysis and test-bed-based detection of vulnerabilities are studies in [14,15]. However, very few of these works report on the detailed cyber threat classification with a particular focus on IEC 61850-based substation, which is the main motivation of this paper.

Although several research methodologies have been proposed to concentrate on the protection of IT systems and networks from various attacks, these protection schemes are unable to guarantee proper security all the time. As a result, the classification of cyber securities, cyber system’s vulnerabilities identification, and the analysis of the system response to the attacks are highly crucial. To diagnose the vulnerabilities of the smart grid, several cyber assessment methods are proposed in the context of different subsystems. These studies help to understand the attack scenarios and system response and thus provide the required information for designing cyber detection/protection systems [16]. In the similar pattern, there is a growing need to analyze existing threats in smart substation which involves data-fusion and signal processing of these devices based on PMU measurements. This also includes their effects on the operation, which defines the scope of this work and the scope of the prestigious MDPI *Sensors* journal.

The main contribution of this paper is to analyze a specific cyber-attack: Denial of Service (DoS) in a digital substation automation system. A comprehensive in-depth analysis for DoS attack in an IEC 61850 architecture is studied with four case scenarios. These attack scenarios are: (1) DoS attack on server, (2) DoS attack on HMI, (3) DoS attack on IEDs, and (4) the effect of varying inter-arrival times. The results of these attacks are then further demonstrated.

**Figure 2:**
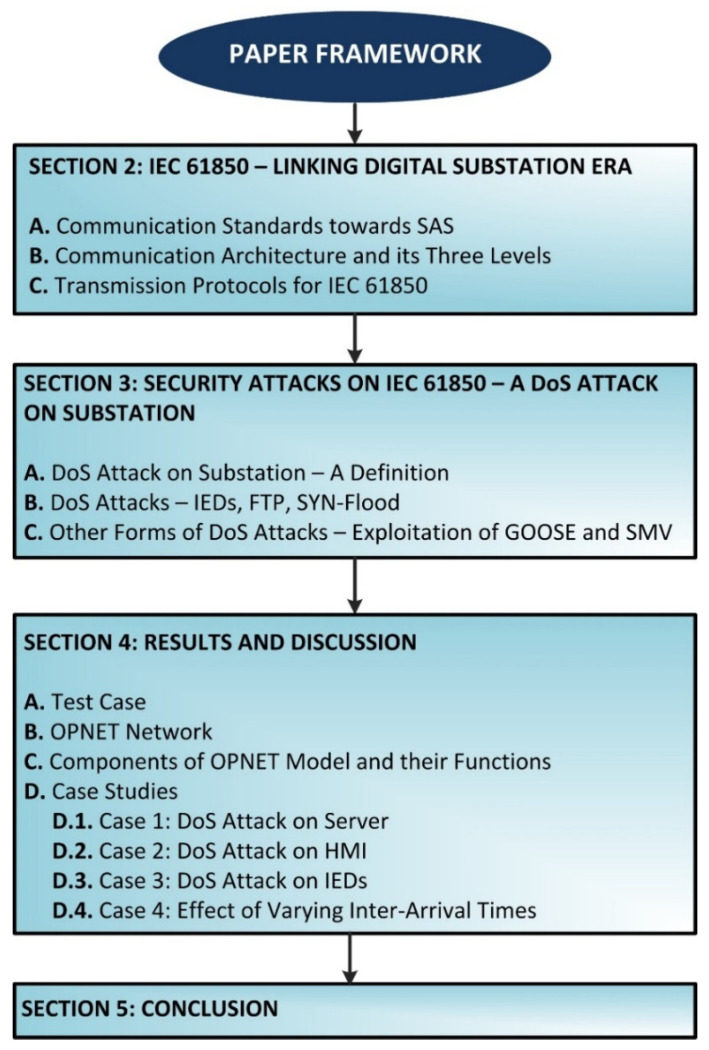
Framework of the paper.

The rest of this paper is organized as follows. Section 2 talks about the linking digital substation era in IEC 61850. Section 3 discusses the possible security attacks in IEC 61850, with a focus on DoS attacks (SYN-FLOOD). Section 4 illustrates the simulation and results which involve the model system and its components. Finally, Section 5 concludes this paper and suggests future research work. Figure 2 shows the complete framework of the paper.

## 2. IEC 61850: Linking Digital Substation Era

This section talks about the communication standards, communication architecture, and transmission protocols of IEC 61850.

### 2.1. Communication Standards towards SAS

The IEC 61850 standard of communication is a globally recognized standard for communication systems in digital substations [17]. The standard focuses on creating a SAS with the objectives of achieving: (1) an open system, (2) lower cost, (3) flexibility, (4) higher efficiency, and (5) expandability [18]. An open system refers to a system that allows for the interoperability of IEDs from different vendors. The IEDs can exchange data between each other and then utilize this information to execute different functions. A lower cost for SAS is achieved by providing an equal playing field for competitors since IEDs from different vendors can be utilized. Every vendor can create and implement their own design to carry out a specific function, data can be exchanged over the communication bus, and individual IEDs are able to be tested without causing any disruptions in the normal operation, hence providing greater flexibility.

**Figure 3:**
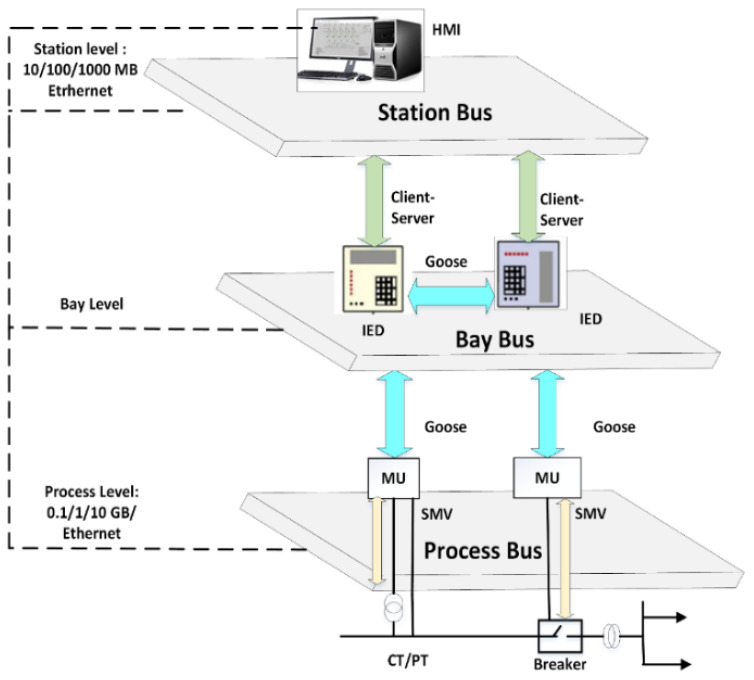
Architecture of IEC 61850 with communication strategy [20].

Maintenance and operation procedures are standardized. Moreover, the use of copper cables is also reduced, which further reduces the cost to implement. Ethernet-based communications which operate at high speeds are used to create a data management system which increases its efficiency and reduces operational delays. Expandability refers to the ability of the communication system to be changed or increased in size with ease as the requirements of the power system change. Since the network is built using Ethernet technology, it is easy to take into consideration any potential changes that may occur in the future. IEC 61850 aims to cover all aspects of substation automation technology by reducing the complexity and maintenance cost, specifying protocols such as TCP/IP and UDP/IP, and ensuring data interoperability among multivendor IEDs (Intelligent Electronic devices) [19]. Figure 3 presents a detailed architecture of IEC 61850 along with communication strategy.

### 2.2. Communication Architecture and Its Three Levels

The IEC 61850 communication architecture consists of three levels: the (1) station, (2) bay, and (3) process levels. Measurement devices such as CT/PT, I/O devices, sensors, and actuators correspond to the process level, whereas the bay level has IEDs, and the station level comprises the Human Machine Interface (HMI) and station controllers.

### 2.3. Transmission Protocols for IEC 61850

Transmission protocols defined in the IEC 61850 standard that are used to deal with data transfer include: (1) the GOOSE protocol, (2) Manufacturing Message Specification (MMS), and (3) Sampled Measured Value (SMV) [21].

Two types of communication services between devices in an SAS are allowed by IEC 61850 [22]. The first type of communication is the client–server model and the second is the peer-to-peer (P2P) model. In the client–server model, the client sends a connection request to the server. This request can either be rejected or accepted. Note that in this communication model, many clients can connect to one server only. The P2P model of communication has no requirement for a centrally located server. This is because each device can operate both as a client as well as a server. Hence, clients can directly connect to each other. The P2P model is used for Generic Substation Event (GSE) services that are time critical events which require reliable and fast communication such as the tripping of a circuit breaker by an IED. GSE services include GOOSE as well as Generic Substation State Event (GSSE) [23]. Unlike GOOSE, in which data in either status or value format is grouped into a data set and then transmitted, GSSE can only transmit status data, and it does so in the form of a status list, which is a string of bits. The SMV protocol is used to transmit instantaneous values of measured power system quantities such as current and voltage. MMS is used to send status information to SCADA for the monitoring of the substation.

**Figure 4:**
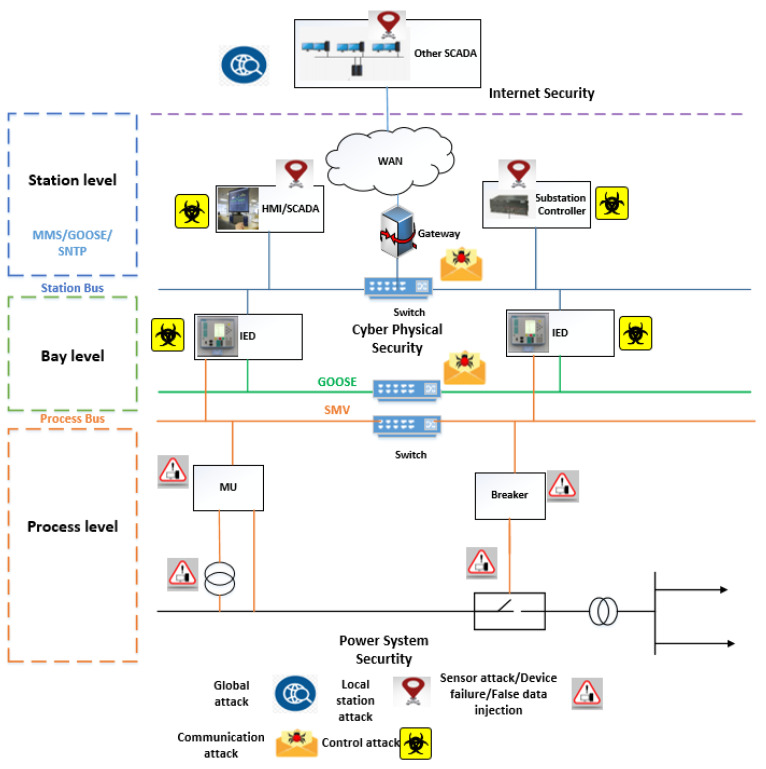
Security threat classification for different levels of IEC 61850 [27].

Table 1 lists the six different types of messages in IEC 61850 [24]. GOOSE messages can be type 1 or 1A while Sampled Values are of type 4. GOOSE and SV use three communication layers of the Open Systems Interconnection (OSI) model. These are: (1) the application layer, (2) the data-link layer, and (3) the physical layer. GOOSE and SV are time-critical messages and are directly mapped to the low-level Ethernet link layer from the application layer [25,26]. The lay out and possible vulnerabilities for IEC 61850-based substation can be seen in Figure 4.

Once the communication standards, three levels of its architecture, and transmission protocols are defined, this leads to the security attacks on IEC 61850.

## 3. Security Attacks on IEC 61850—A DoS Attack on Substation

Security attacks are defined as a set of any attacks to a communication network, which could control, crush, sabotage, modify, hack, or access network’s data without proper permission from the authority. Being an Ethernet-based technology, an IEC 61850-based substation is prone to cyber-attacks and can be a victim of such malicious security threats. According to [28], there are numerous types of cyber-attacks that can be used to disrupt the operation of the smart grid. The possible attacks on IEC 61850-based systems are summarized in Table 2. The attacks are primarily classified into two categories: (1) attacks on the network and (2) attacks on the messages. Attacks on the network can be categorized into several groups. Each group is listed in Table 2 with references. Attacks on the messages can be modified or exploited by the hackers and can cause disruption in the network.

### 3.1. DoS Attack on Substation—A Definition

A DoS attack is classified as a security attack where the hackers/attackers attempt to prevent legitimate users or machine of a specific service from accessing that service. This attack is generated by distributing false instructions to that server or service. In this process, the victim system/server is flooded with excessive requests by the hackers, causing overloading or unresponsiveness of the system/service, and authorized user requests are denied by the system [39]. Similarly, when hackers employ several machines and communication connections to flood the victim system, it is known as a Distributed Denial-of-Service (DDoS) [40], which has a more severe impact on the system.

### 3.2. DoS Attacks—IEDs, FTP, SYN-Flood

**Figure 5:**
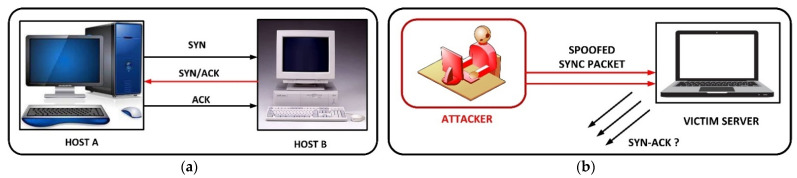
SYN-FLOOD attack: (**a**) three-way successful handshake and (**b**) attack mechanism.

In the literature, there have been several reports on DoS attacks focused on IEC 61850-based substations. In [41], the authors demonstrated DoS attacks using common services of IEDs such as File Transfer Protocol (FTP) and telnet on port number 23, which eventually have kept the targeted system idle all the time. The other types of DoS attacks that have been reported in the literature are SYN-Flood attack and buffer overflow attack [42,43]. The SYN-Flood attack mode is introduced, which exploits vulnerabilities in TCP protocol to launch an attack. The TCP three-way handshake is a technique used by the TCP protocol to establish a connection between devices. A simplified overview of this process is host A sends a TCP synchronize packet to host B. Upon receiving the packet, Host B sends a synchronize acknowledgement back to host A. Host A then sends an acknowledgement back to host B, thereby establishing a TCP connection. This process is often referred to as (SYN, SYN-ACK, ACK) [44]. In the SYN-Flood attack, hackers can send SYN packets to multiple ports of a targeted server using fake IP addresses. The receiving device believes it is obtaining legitimate connection requests and hence it tries to respond to each of the requests by sending back SYN-ACK packets from each of the targeted ports. This packet is never able to reach back to the sending device because the IP address is fake and hence the receiving device will never receive an ACK packet to establish the connection. The receiving device is not able to close the connection, and before the connection is able to timeout, another SYN packet will be received by the server. This causes a lot of semi-open or half-open connections to be present at the same time. Once the number of open connections surpasses the capacity of the server, it will deny connections including from legitimate requests, and it may also cause the server to crash. A typical SYN-FLOOD is demonstrated in Figure 5, where the victim server is unable to establish authorized connection where the attack modes are represented.

### 3.3. Other Forms of DoS Attacks—Exploitation of GOOSE and SMV

There are other forms of DoS attacks discussed in the literature [45,46], which exploits GOOSE and SMV. In this attack scenario, the IEDs start malfunctioning because of the large number of GOOSE or SV messages transmitted by the attackers and causing the normal operation of them. Another form of DoS is the GOOSE poisoning attack described in [47], where authorized GOOSE messages are denied by the subscriber IEDs due to the injection of the false GOOSE messages by the attackers. The attackers employ high status number attack, high-rate flooding attack, and semantic attack in order to perform a GOOSE poisoning attack. However, the discussion of this paper will be focused on the SYN-Flood attack mode.

## 4. Results and Discussion

### 4.1. Test Case

**Figure 6:**
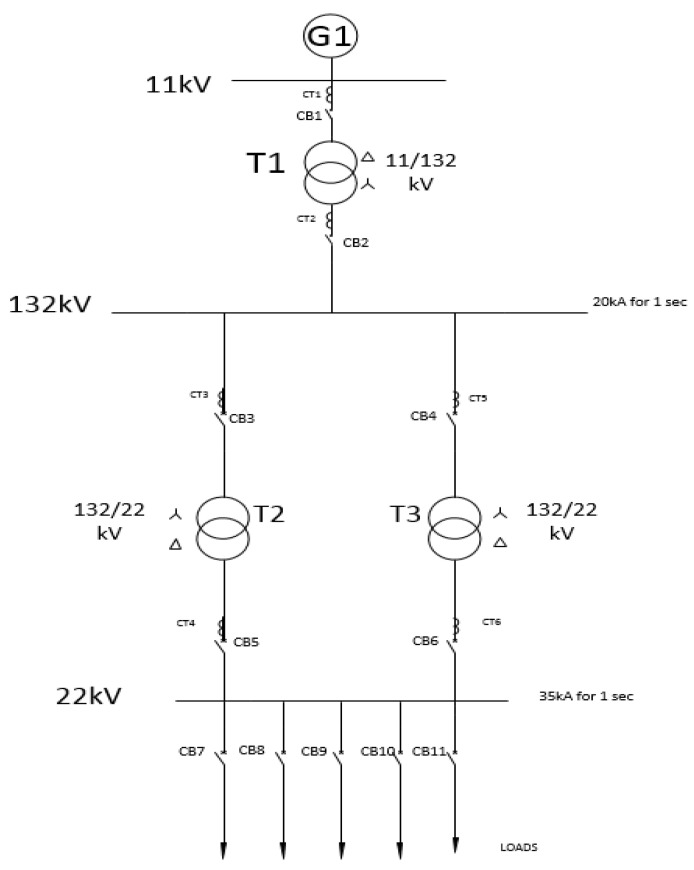
Power system layout.

The test case involves simulating a section of the power grid in a substation. This can also be seen in Figure 6. Transformer 1 (T1) between bus bars B1 and B2 can be taken as the substation that will be simulated. CB1 and CB2 are connected to IEDs in the bay level which control, monitor, and protect the power system. Power is generated at G1 at a base level of 100 MVA and a voltage of 11 kV, as shown at B1. T1 steps up the voltage to 132 kV, as can be seen at bus bar 2 (B2). Two parallel feeders are present from the B2 to the 22 kV bus (B3) with Transformers T2 and T3 on separate lines. All the transformers within the system are protected using differential protection through the current transformers.

### 4.2. OPNET Network

The Optimized Network Engineering Tool (OPNET) is a powerful networking tool used to run simulations of complex communication networks. It is a network simulation tool set consisting of various products and modules for different purposes. The product modules used for the purpose of this project are the OPNET Modeler and OPNET Modeler Terrain Modelling. The OPNET Modeler is a discrete event simulator with an inbuilt graphic user interface (GUI). It can run analytical simulations, hybrid simulations, as well as 32- and 64-bit parallel simulations.

### 4.3. Components of the OPNET Model and Their Functions

**Figure 7:**
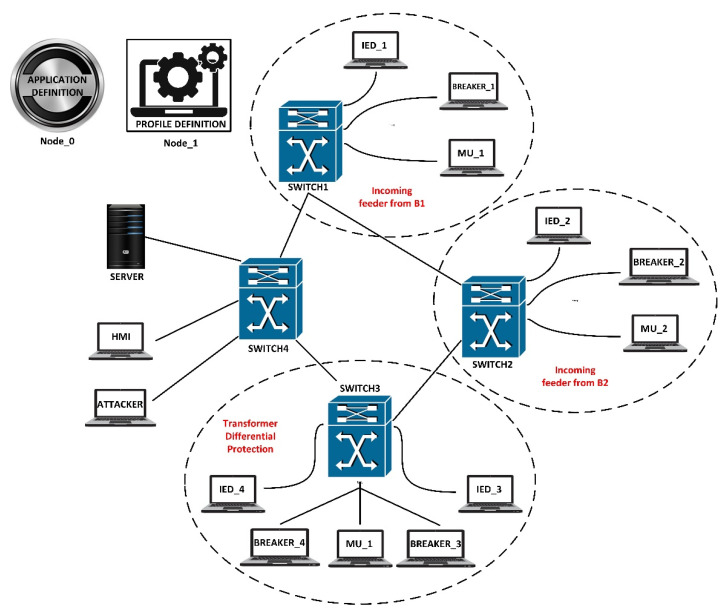
OPNET-based power grid substation layout.

The OPNET model includes four switches. These switches are placed in a ring network topology with Switches 1 and 2. Both these switches have an IED node, Breaker Node, and Merging Unit (MU) nodes attached to them. With reference to the power system layout described in Figure 7, the functionality of these components is described as follows. IED 1 provides protection and control to the incoming feeder from bus bar (B1) into the substation. MU_1 provides measurements taken from the process level of the substation which the IED analyses and may take necessary action if required. Breaker_1 represents the circuit breaker on the incoming feeder between T1 and G1. Switch 2 is also connected to three ethernet station modules representing an IED, a breaker, and an MU. Breaker_2 represents the circuit breaker on the outgoing feeder from substation T1 to B2. Switch 3 has 5 ethernet nodes attached which include IED’s 3 and 4, breakers 3 and 4, as well as a merging unit. The purpose of these nodes is to provide differential protection of the substation transformer T1. Switch 4 is the last switch in the network. The station HMI and server are connected to this switch. The application and profile configuration nodes shown below have been used to create background traffic flows in IEC 61850-compliant digital substations.

### 4.4. Case Studies

**Figure 8:**
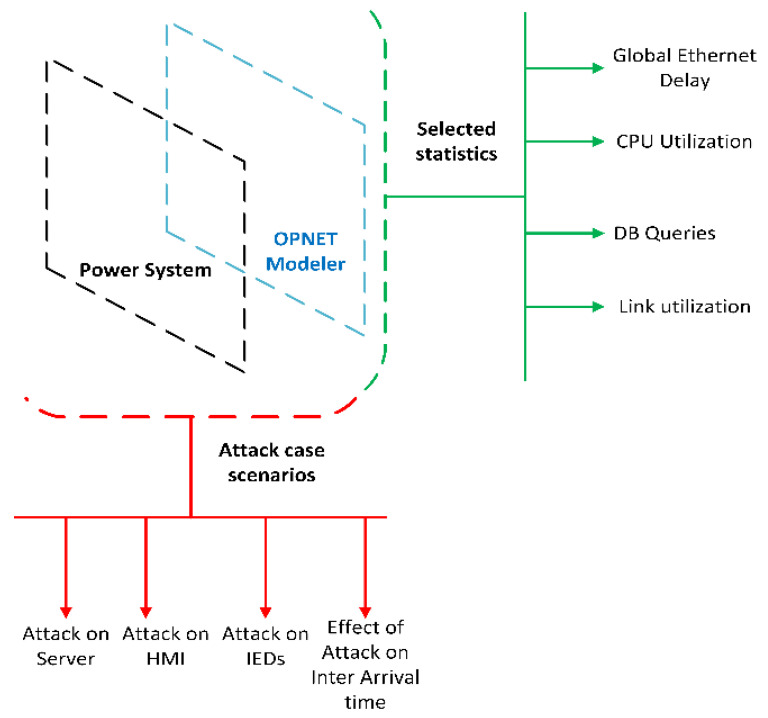
Simulation and model system framework.

The case studies and simulations have been performed in the IEC 61850 laboratory of Curtin University. In this section, an OPNET model will be utilized to analyze the four vital statistics for further analyzing the attack cases. Table 3 provides the definition of these selected statistics. In Figure 8, the simulation and model system are illustrated where the attack cases and selected statistics are presented.

#### 4.4.1. Case 1: DOS Attack on Server

**Figure 9:**
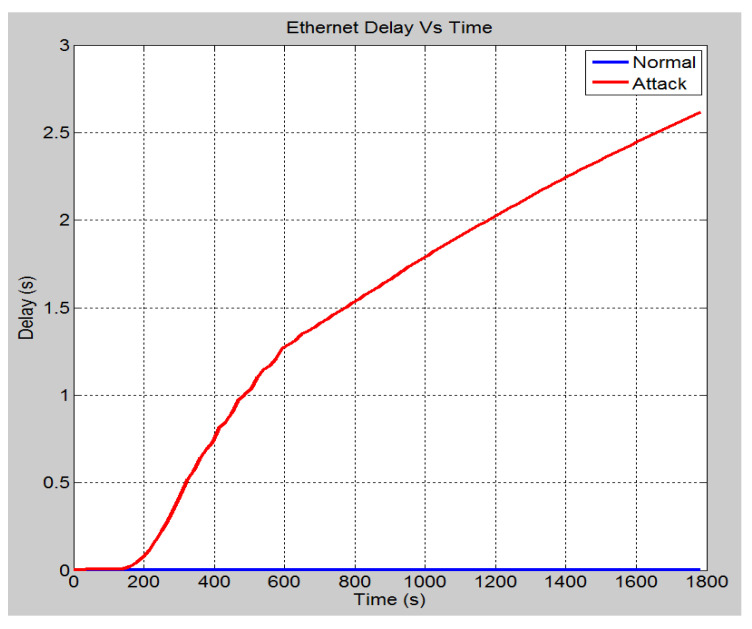
Average global ethernet delay (s).

**Figure 10:**
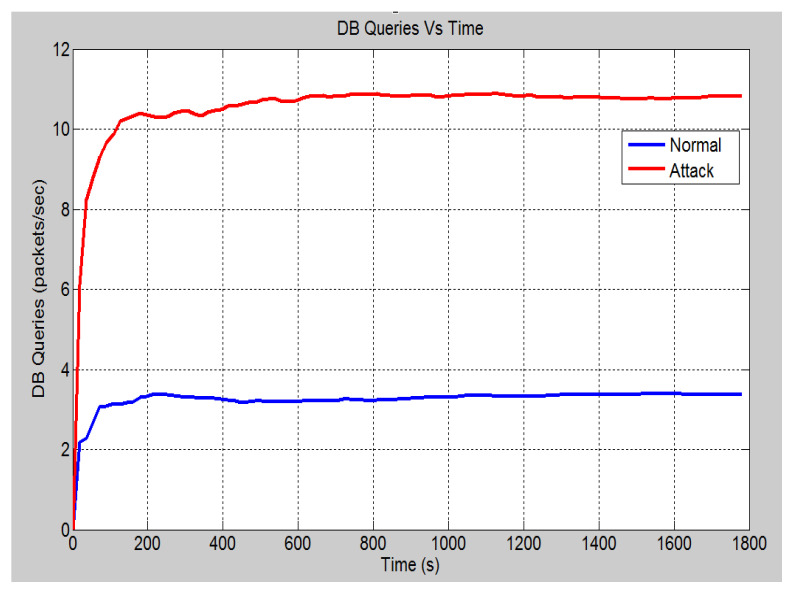
DB queries.

**Figure 11:**
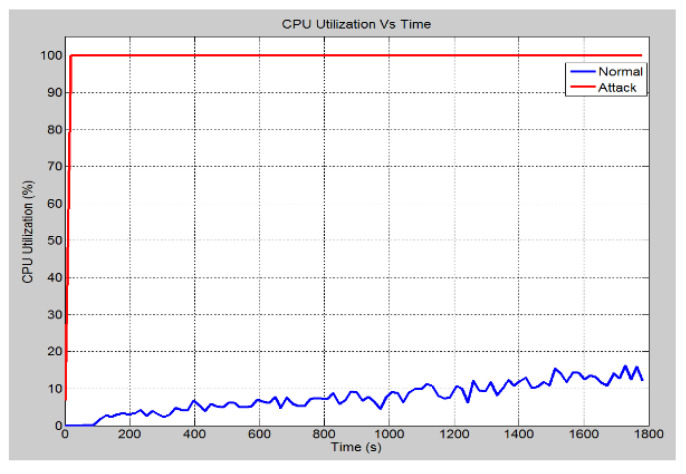
CPU utilization rate (%).

This scenario talks about the assumption where a hacker gains access to the bay level into switch 4 (See Figure 6 for power system layout). This access was made through the attacking node, which is connected to port 12 of switch 6. In such a situation, from this device, an attack is launched on the port by flooding the receiving ports with data. This is done by disguising the source IP address and setting it to an unreachable address. Figure 9 demonstrates the effect of the SYN-Flood DoS attack on the global ethernet delay in the system. It shows that in the no attack scenario, the average Ethernet delay is 0.57 milliseconds. However, once the attack is launched, the ethernet delay rises to 2.6 s at the 30 min mark and will continue to increase until the server crashes. Note that for a GOOSE message relating to the trip operation, the maximum allowable delay is 1 millisecond. Hence, the attack has effectively nullified the ability of the system to implement the required operations within the acceptable limits. Figure 10 demonstrates the number of database queries sent to the server in both scenarios. Under normal operating conditions, the server receives slightly fewer than 3.5 packets per second. In the attack scenario, the number of queries received is 11 packets per second. This shows that the number of incoming queries increased by a factor of three. Figure 11 demonstrates the average CPU utilization for both scenarios. Under the normal operating conditions, the maximum CPU usage of the server is 16.2% of its maximum capacity. Once the DOS attack is launched, the average CPU utilization increases to 100% of its maximum capacity within 1 min of the attack. The attack has occupied the server with false data and has severely limited its ability to establish connections with legitimate clients and therefore causing the DoS.

#### 4.4.2. Case 2: DoS Attack on HMI

**Figure 12:**
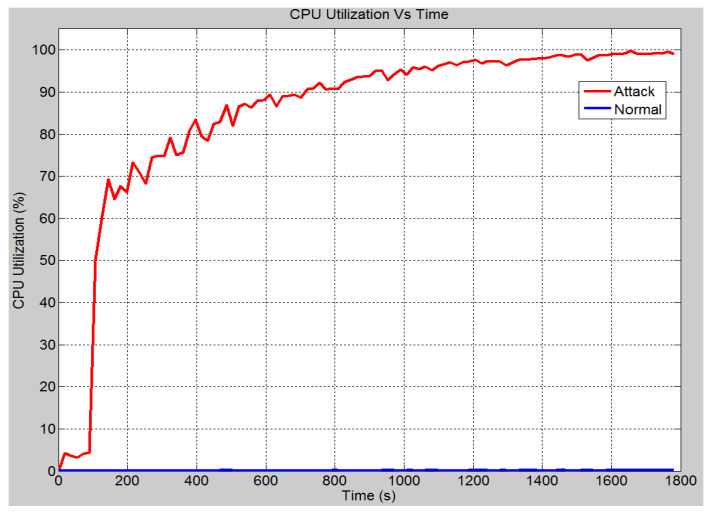
HMI CPU utilization rate (%).

**Figure 13:**
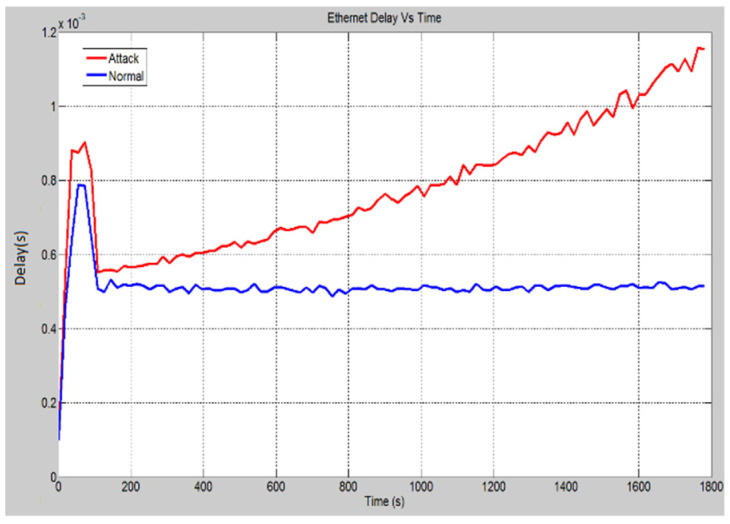
Global Ethernet delay (s) for the entire system.

In this scenario, a DoS attack has been launched on the HMI computer. This is to prevent personnel from executing the control and monitoring functions. Figure 12 demonstrates the CPU utilization of the HMI computer under both conditions. Under normal operation, the CPU utilization remains close to 1%. However, once the attack has been launched, it has been noticed that the CPU utilization rises significantly. At approximately 15 min, the CPU utilization has been increased to over 95% and ends at 100% CPU utilization. As a result, operators have lost their access to carry out control and protection operation through the IEDs. This is because the computer has been occupied with all the false messages it has received. In the same pattern, the global Ethernet delay for the entire system is shown in Figure 13. The Ethernet delay is higher in the attack scenario. However, the difference in the delay between the two scenarios is approximately 0.7 milliseconds. The Ethernet delay in the attack scenario is within the acceptable limit. The reason for the marginal increase in the Ethernet delay can be attributed to the fact that the number of requests received by the HMI station is significantly lower than that received by the server as in the previous scenario. In other words, the other nodes are not as reliant on the HMI station as they are on the ethernet server. Hence, causing the HMI to crash does not cause the Ethernet delay to increase as significantly. In addition to that, the other IEDs are working properly and are not suffering any sort of malfunction hence the operating conditions are ideal and a low Ethernet delay is expected.

#### 4.4.3. Case 3: DoS Attack on IEDs

**Figure 14:**
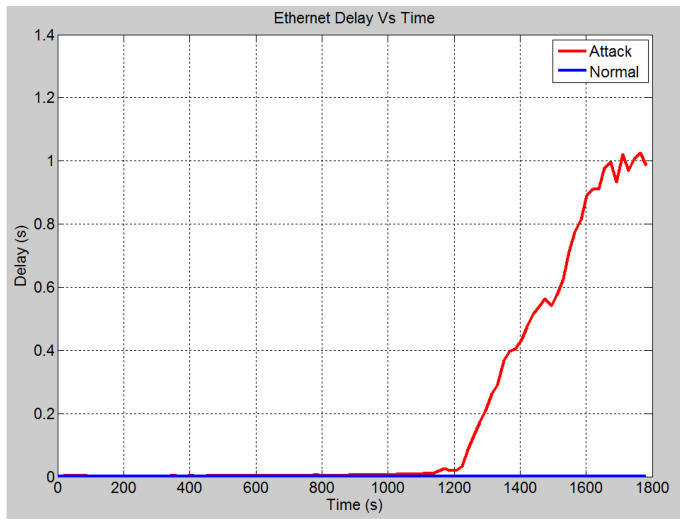
Case 3: Global Ethernet delay (s) in no-attack scenario.

**Figure 15:**
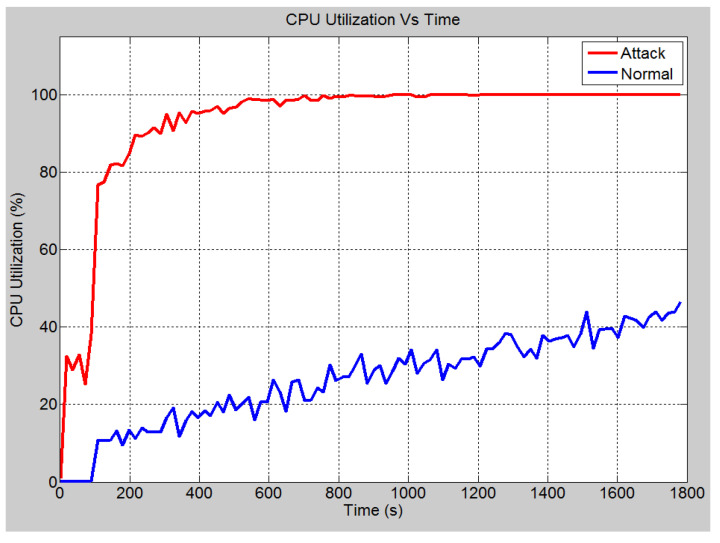
Case 3: IED CPU utilization rate (%).

In this scenario, a DoS attack is carried out on both IEDs 3 and 4. This is also shown in the OPNET layout in Figure 7. The attackers are able to manipulate the data input into the IEDs and overburden the CPUs and consume the link bandwidth. The following results demonstrate the effect of such attack. In the no attack scenario, the global Ethernet delay is approximately 0.52 milliseconds (See Figure 14). This means that GOOSE messages are sent within an acceptable time limit. Under the attack scenario, the Ethernet delay rises to approximately 1 s. The CPU utilization under the two different scenarios is compared in Figure 15. Under normal operation, a maximum CPU usage of approximately 46% is observed while under the attack conditions, the CPU usage raises to 100% within 10 min approximately. The significant increase in the CPU utilization for the attack scenario means that the IED is unable to respond to legitimate requests and hence denial of service has been occurred.

#### 4.4.4. Case 4: Effect of Varying Inter-Arrival Times

**Figure 16:**
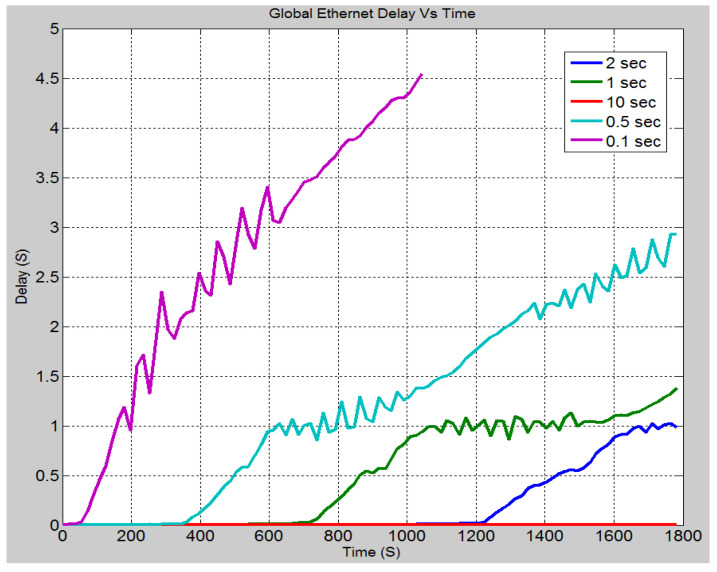
Case 4: Global Ethernet delay (s) for different inter arrival time.

**Figure 17:**
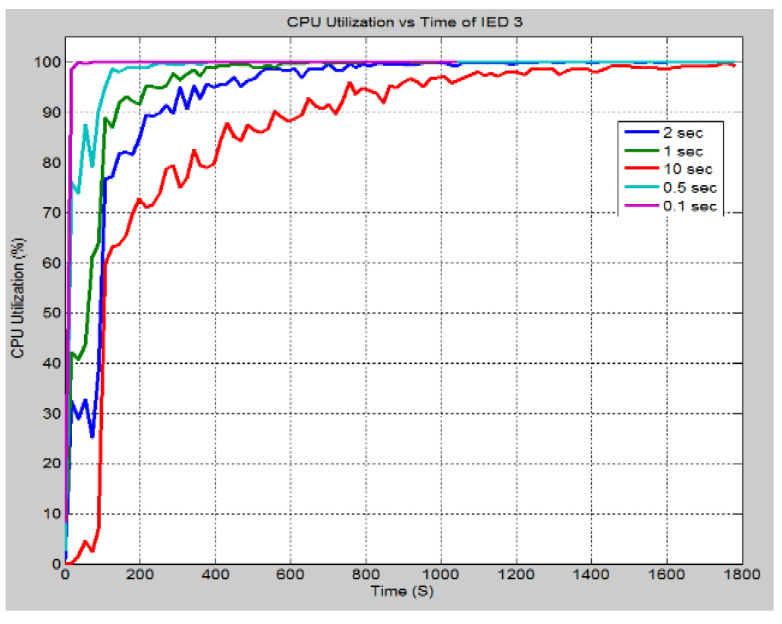
Case 4: CPU Utilization rate (%) for different inter arrival time.

**Figure 18:**
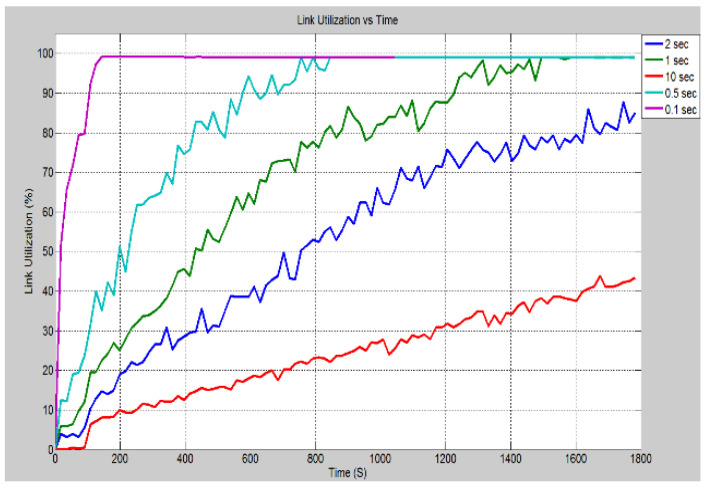
Case 4: Link Utilization (%) for different inter arrival time.

**Figure 19:**
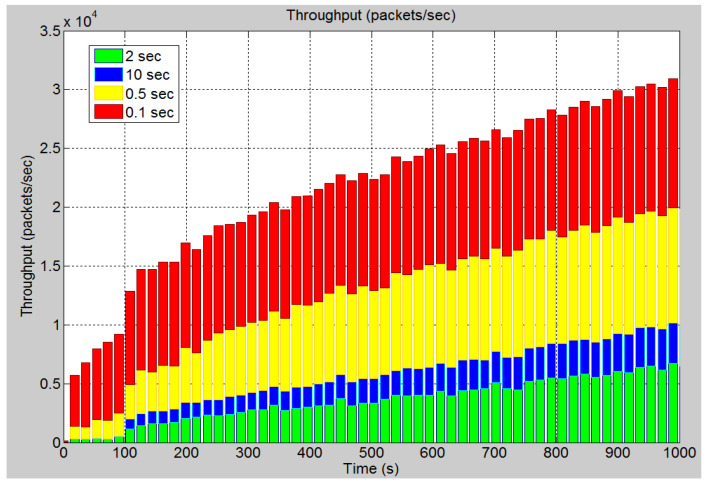
Case 4: Throughput (packets/s) for different inter arrival time.

In this scenario, the rate at which the attack data has been sent to IEDs 3 and 4 is altered. This was to observe the effect on the system. Figure 16 demonstrates the effect of changing the inter arrival times of the attack data, i.e., the rate at which the data is sent. As can be seen when the inter-arrival time is set to 0.1 s, the delay is significantly greater, and it increases at a much faster rate as determined by the greater slope. As the inter-arrival time is increased, the delay becomes smaller, and it takes longer for the attack data to cause the system delay to increase. This is expected since the more data the device receives in a short period, the longer it takes to process, and hence delays are increased. In Figure 17, it can be seen that the CPU utilization rate takes longer time to reach 100% as the inter-arrival time is increased. With an inter-arrival time of 0.1 s, there is an almost instantaneous jump to full CPU utilization, while the inter-arrival time of 10 s takes longer time. The higher the inter-arrival time is, the more time it takes to reach full CPU utilization. Figure 18 demonstrates the link utilization for the different inter-arrival times. As expected with the faster inter-arrival time, the link reaches to its maximum capacity much faster. With the 0.1 s inter-arrival time, 100% link utilization is achieved within 36 s. However, with the 1 s inter arrival time, 99% link utilization is achieved in 1494 s. Additionally, it can be seen that the 10 s inter-arrival time does not reach at 100% link utilization. By conducting the simulation for a longer duration, a greater link utilization would have been achieved. However, 100% link utilization does not appear to be feasible. The reason for this is the recipient IEDs will begin to drop the pending connections due to the long waiting time between requests. Figure 19 demonstrates the effect of changing the inter-arrival time of the attack data on the throughput between IED 3 and switch 3. From the stacked bar chart, it is evident that the lower inter arrival time results in more packets per second through the link resulting in higher link utilization. An inter-arrival time of 0.1 s has the highest throughput while a 10 s inter-arrival time has the lowest link throughput.

## 5. Conclusions

The proposed communications network and the simulation results intend to provide a whole scenario of the DoS attack on the SAS. The results are especially helpful and significant for the preliminary understanding of the effects on the performance of a digital substation. The major statistics that were observed during these experiments are the global Ethernet delay, link utilization, CPU utilization, and link throughput, respectively which involves communication, signal, and data processing/fusion in the network, which are also the scope of the prestigious MDPI Sensors Journal. It can be concluded that an attack on the server results in a larger ethernet delay as compared to an attack on the HMI or IEDs. The IEC 61850 standard communication protocols of GOOSE and SMV messages are prevented by the DoS attack from being transmitted to its destination. The results indicated that the inter arrival time of the attack data plays a significant role in the delays and CPU utilization in the system. A faster inter-arrival time results in maximum CPU usage in comparison with a slower inter-arrival time. Further research in this area will focus on the implementation of different types of coordinated cyber-attacks on the system. This will also include various counter measures to prevent those attacks, including implementation of a real-time testbed utilizing Ethernet switches, different physical IEDs from vendors, and an OPAL RT real-time simulator.

## Figures and Tables

**Table 1:** Transmission protocols for IEC 61850.

Communication Architecture	Service	Message Type	Application Type	Time Requirement (Milli-Second)	Communication Mapping
Client–Server (SCADA)	ACS	2	Moderate speed	100	Ethernet TCP/IP
		5	File Transfer	>=1000	
Publisher–subscriber	GOOSE, GSSE	1A	Trip	3–100	Ethernet
		1B	Others	20–100	
	SMV	4	Measurement data	3–10	Ethernet
	TS	6	Synchronization	N/A	Ethernet UDP/IP

**Table 2:** List of attacks on IEC 61850-based SAS.

Attack on IEC 61850	Types of Attack	Action on the SAS	Effects on SAS	Ref.
**Security Attacks on IEC 61850 Network**	Malformed Packet Attack	Transmits malformed packets to IEDs	Communication failure among the IEDs	[29]
	DoS (Denial of Service) Attack	Floods the targeted IED with false messages	Consumes link bandwidth and increase the CPU utilization rate	[30,31]
	Address Resolution Protocol (ARP) Spoofing Attack	Fools a receiver into thinking it is being communicated to by a trusted source	IED will communicate with the attacker’s laptop instead of SCADA	[32]
	Man in the Middle (MITM) Attack	An attacker in the substation level redirects communication traffic between the IED and SCADA to a malicious laptop	Malicious control commands are sent remotely and changes the protection settings of IEDs	[33,34]
	Configuration Tampering	Alters the configured IED description (CID) file within the IED	Disruption of the communication protocols and monitoring system	[35]
**Security Attacks on IEC 61850 Messages (exploitation of GOOSE and SMV)**	GOOSE and SV Modification Attack	The content of the captured network packets are modified	IEDs can be accessed by hackers and can be a victim of performing malicious acts	[36]
	GOOSE and SV DoS Attacks	Attacker sends oversized or large number of GOOSE and SMV in the network	Failure of the IEDs to respond to authorized users	[37]
	GOOSE and SV Replay Attack	Attacker captures network packets transmitted among the hosts and replays back without any change in the message to obtain a similar response	Causes false tripping of the breaker and can lead to catastrophic situation	[38]

**Table 3:** Analysed statistics for system performance.

Statistics Name	Definition
Global ethernet delay [48]	The global ethernet delay statistic is used to demonstrate the end-to-end delay of all the packets that are received by every station. In other words, it represents the time taken for a packet to travel from the source to the destination.
CPU utilization [49]	The CPU utilization statistic is used to show the CPU usage of a particular node in the network. The CPU usage models the IP packet forwarding delays and application processing delays.
Data-base queries [50]	A DB query is an inquiry by a client device to the server (database) to obtain information in a manner that can be read. In the results, the DB query statistic is measured in terms of traffic received in packets per second.
Link Utilization [51]	The link utilization statistic displays the percentage of the available channel bandwidth being consumed by the flow of traffic within the network.

## Data Availability

Not Applicable.
